# Association of Estimated Plasma Volume Status With Invasive Hemodynamics and All‐Cause Mortality in Patients With Liver Cirrhosis

**DOI:** 10.1002/jgh3.70195

**Published:** 2025-06-02

**Authors:** Esteban Kosak Lopez, Phuuwadith Wattanachayakul, Jose Manuel Martinez Manzano, Andrew Geller, Simone A. Jarrett, John Malin, Raul Leguizamon, Tara A. John, Rasha Khan, Ian McLaren, Alexander Prendergast, Kevin Bryan Lo, Zurab Azmaiparashvili

**Affiliations:** ^1^ Department of Medicine; Jefferson Einstein Philadelphia Hospital Sidney Kimmel Medical College Philadelphia Pennsylvania USA; ^2^ Division of Pulmonary and Critical Care Medicine Brigham and Women's Hospital, Harvard Medical School Boston Massachusetts USA; ^3^ Gastroenterology & Hepatology Division NYU Grossman School of Medicine New York USA; ^4^ Division of Cardiovascular Medicine Brigham and Women's Hospital, Harvard Medical School Boston Massachusetts USA

**Keywords:** cardiac catheterization, estimated plasma volume status, hemodynamics, liver cirrhosis, liver transplantation

## Abstract

**Background:**

Estimated plasma volume status (ePVS) correlates with intravascular congestion and prognosis in patients with heart failure. The ePVS relationship with invasive hemodynamic profiling and clinical outcomes in patients with liver cirrhosis (LC) remains unclear.

**Methods:**

This single‐center retrospective cohort study included LC patients who underwent right heart catheterization (RHC) between 2018 and 2023. Estimated plasma volume status (ePVS) was calculated using the Strauss‐derived Duarte formula, with patients classified into high (> 5.5%) and low‐ePVS (≤ 5.5%) groups. Cox‐multivariable analysis was used to determine if ePVS was associated with all‐cause mortality within 1 year post‐RHC among transplant‐free patients.

**Results:**

Of the 353 patients with LC (median age 59 years, 59% male, 45% Caucasian, and 29% African American), 79% were classified into the high‐ePVS group. Compared to the low‐ePVS group, the high‐ePVS group had significantly higher right atrial pressure (9 vs. 6 mmHg, *p* = 0.01), pulmonary arterial wedge pressure (14 vs. 11 mmHg, *p* = 0.014), cardiac output (9.8 vs. 6.4 L/min, *p* < 0.0001), and cardiac index (5 vs. 3.1 L/min/m^2^, *p* < 0.0001). Additionally, the high‐ePVS group exhibited a higher prevalence of cirrhosis‐related complications, including ascites, splenomegaly, and varices, and a greater likelihood of receiving orthotopic liver transplantation within 1 year (38% vs. 11%, *p* < 0.0001). Among transplant‐free patients, ePVS was independently associated with all‐cause mortality at 1 year (HR 1.15, 95% CI: 1.00–1.32, *p* = 0.048).

**Conclusion:**

Our study demonstrated that ePVS was associated with intravascular congestion, hyperdynamic circulation, and cirrhosis complications. Furthermore, ePVS was independently associated with all‐cause mortality among transplant‐free LC patients.

## 1 Introduction

Liver cirrhosis (LC) is among the leading causes of mortality in the United States and often leads to complications arising from portal hypertension and hepatic dysfunction, including ascites, variceal bleeding, hepatic encephalopathy, renal dysfunction, and hepatocellular carcinoma. These complications markedly impair the quality of life and reduce survival [1].

LC patients frequently experience hyperdynamic circulation and increased intravascular congestion, both increasing portal blood inflow and pressure, further adding complexity to disease management [2]. Recent studies indicate that estimated plasma volume status (ePVS) may be a valuable marker of intravascular congestion [3], showing prognostic significance in patients with heart failure (HF) through its association with fluid overload and adverse clinical outcomes [4].

While ePVS has shown utility in HF and other cardiovascular conditions [5], its application in LC remains less defined, particularly regarding adverse outcomes and congestion severity as indicated by echocardiographic and right heart catheterization (RHC) findings. Investigating ePVS in LC patients may help clarify the relationship between fluid balance and liver disease severity, offering insights into its prognostic potential and value in guiding management strategies.

Accordingly, we aim to evaluate the correlation between ePVS and hemodynamic parameters derived from RHC. Additionally, we sought to examine the association of ePVS with all‐cause mortality within 1 year following RHC.

## 2 Methods

We conducted a single‐center retrospective cohort study of LC patients who underwent right heart catheterization (RHC) between August 2018 and June 2023 and were followed up for 1 year after the RHC date. Patients undergoing RHC were identified using the current procedural terminology (CPT) codes [6]. The diagnosis of LC was identified by chart review of existing clinical documentation, on the basis of known medical history of cirrhosis and abdominal imaging reports with features consistent with cirrhosis (including hepatic surface nodularity, hepatic atrophy, and signs of portal hypertension). Only patients with available hemoglobin and hematocrit (H&H) values available within 1 week before or after RHC were included.

Baseline demographics, RHC reports, liver cirrhosis features (causes and complications), laboratory data, liver transplant status, and all‐cause mortality were collected retrospectively from institutional electronic medical records. RHC indications were based on common clinical scenarios in liver cirrhosis, including liver transplant (LT) evaluation, valvular heart disease, hemodynamic and volume status assessment in patients with fluid overload symptoms (ascites or edema), and suspected pulmonary hypertension due to abnormal echocardiographic findings. The primary outcomes included all‐cause mortality at 30 days and 1 year among transplanted and transplant‐free LC patients.

The Strauss‐derived Duarte formula was used to calculate the estimated plasma volume status (ePVS) as follows: ePVS = 100 × (1—hematocrit)/hemoglobin [7]. Data were stratified into high‐ePVS (> 5.5) and low‐ePVS (≤ 5.5) groups according to prior heart failure studies [8]. Cardiac output (CO) and cardiac index (CI) were determined using the Fick principle. Liver disease severity was assessed using the Model for End‐stage Liver Disease‐Sodium (MELD‐Na) score [9]. Abdominal imaging (ultrasonography, computed tomography, or magnetic resonance imaging) were reviewed to assess the presence of portal venous circulation abnormalities (including increased portal vein diameter, decreased blood flow, and portosystemic collaterals), ascites, and splenomegaly. The study data were collected using the Jefferson REDCap database. This study was approved by Jefferson's Institutional Review Board (iRISID‐2023‐2277).

Categorical data were summarized using frequencies and percentages, while continuous variables were presented as medians with interquartile ranges (IQR) owing to skewed distributions. Bivariate analyses used *χ* ^2^ or Fisher's exact tests for categorical variables, as appropriate, and Wilcoxon rank‐sum tests for continuous variables. Kaplan–Meier curves and log‐rank tests compared 1‐year all‐cause mortality between the low‐ and high‐ePVS groups among transplanted and transplant‐free patients. To examine whether ePVS was independently associated with 1‐year all‐cause mortality among transplant‐free patients, we performed a multivariable Cox regression analysis, adjusting for pre‐specified variables, including age, sex, serum albumin, and liver disease severity (MELD, incorporating serum creatinine, total bilirubin, and international normalized ratio [INR]). Lastly, a sensitivity analysis was done to determine the optimal ePVS cutoff in this population using Youden's index method. Hazard ratios (HR) with confidence intervals (CI) were reported, with statistical significance set at a *p* value of 0.05. All analyses were conducted using the Stata software (StataCorp. 2023, Stata Statistical Software: Release 18, College Station, TX, USA).

## 3 Results

### 3.1 Baseline Characteristics

Of 415 LC patients, 353 were included in the analysis. We excluded 62 subjects; 44 were on dialysis, and 18 were without H&H available within a week before or after RHC (Table 1). The median age was 59 years (IQR 52–66), and the patients were predominantly male (59%, *n* = 210) and white (45%, *n* = 158). The most common documented cause of cirrhosis was alcohol consumption (58%, *n* = 205), followed by viral hepatitis (20%, *n* = 69). The median MELD and MELD‐Na scores were 22 (IQR 12–31) and 24 (IQR 15–32), respectively. Seventy‐nine percent (*n* = 281) of the patients had high‐ePVS (> 5.5), and 21% (*n* = 72) had low‐ePVS (≤ 5.5).

**TABLE 1:** Data stratified by estimated plasma volume status (high vs. low ePVS).

Variables	Low ePVS (≤ 5.5) (*n* = 72)	High ePVS (> 5.5) (*n* = 281)	Total (*n* = 353)	*p*
Age in years—median (IQR)	62 (57–69)	58 (50–65)	59 (52–66)	0.0004
Male sex—*n* (%)	51 (71)	159 (57)	210 (59)	0.028
Race—*n* (%)				0.005
White	30 (42)	128 (46)	158 (45)	
African American	29 (40)	72 (26)	101 (29)	
Hispanic	9 (13)	26 (9)	35 (10)	
Asian	2 (3)	10 (4)	12 (3)	
Other	2 (3)	45 (16)	47 (13)	
Body mass index—kg/m^2^	28 (25–32)	29 (25–34)	29 (25–34)	0.305
Right heart catheterization data—median (IQR)				
Right atrial pressure—mmHg	6 (3–12)	9 (5–14)	8 (4–14)	0.01
Mean pulmonary artery pressure—mmHg	22 (15–34)	23 (17–31)	23 (16–31)	0.887
Pulmonary artery wedge pressure—mmHg	11 (6–20)	14 (9–21)	14 (9–21)	0.014
Cardiac output—L/min	6.4 (4.1–8.1)	9.8 (7.4–13.1)	8.9 (6.7–12.3)	< 0.0001
Cardiac index—L/min/m^2^	3.1 (2.1–4.1)	5 (4–6.5)	4.6 (3.4–6.1)	< 0.0001
Pulmonary vascular resistance—wood units	1.6 (1–3.1)	0.8 (0.5–1.3)	0.9 (0.5–1.6)	< 0.0001
Comorbidities and cirrhosis complications—*n* (%)				
Alcohol use	25 (35)	180 (64)	205 (58)	< 0.0001
Viral hepatitis	25 (35)	44 (16)	69 (20)	< 0.0001
Metabolic‐associated liver disease	8 (11)	46 (16)	54 (15)	0.359
Autoimmune hepatitis	3 (4)	10 (4)	13 (4)	0.733
Ascites (*n* = 327)	29 (54)	233 (85)	262 (80)	< 0.0001
Splenomegaly (*n* = 327)	26 (48)	183 (67)	209 (64)	0.008
Portal venous circulation abnormalities (*n* = 327)	33 (61)	216 (79)	249 (76)	0.005
History of varices	13 (19)	118 (42)	131 (37)	< 0.0001
History of TIPS	3 (4)	11 (4)	14 (4)	1
Diabetes mellitus	28 (39)	90 (32)	118 (33)	0.271
Hypertension	49 (68)	149 (53)	198 (56)	0.022
Hyperlipidemia	43 (60)	95 (34)	138 (39)	< 0.0001
Laboratory analysis—median (IQR)				
Hemoglobin—g/dL	13 (12.3–14.7)	8.4 (7.6–9.7)	9.1 (7.7–11.2)	< 0.0001
Hematocrit (%)	39 (37–45)	26 (23–30)	27 (24–34)	< 0.0001
ePVS (range)	4.7 (2.9–5.5)	8.9 (5.6–13.2)	8 (2.9–13.2)	< 0.0001
Platelet count	162 (108–208)	102 (60–146)	108 (64–166)	< 0.0001
Serum creatinine—mg/dL	1.04 (0.8–1.3)	1.5 (0.9–2.6)	1.4 (0.9–2.3)	< 0.0001
International normalized ratio	1.2 (1.1–1.4)	2 (1.5–2.5)	1.8 (1.3–2.4)	< 0.0001
Total serum protein—g/dL	6.9 (6–7.5)	6 (5.3–6.5)	6 (5.4–6.8)	< 0.0001
Serum albumin—g/dL	3.3 (2.9–3.9)	2.9 (2.4–3.5)	3 (2.4–3.6)	0.001
Serum sodium—mEq/L	139 (136–142)	136 (132–139)	137 (133–139)	< 0.0001
Total bilirubin—mg/dL	1.2 (0.7–2)	5.7 (1.9–17)	3.8 (1.2–13)	< 0.0001
Model for end‐stage liver disease	9 (6–14)	25 (16–33)	22 (12–31)	< 0.0001
Model for end‐stage liver disease—Na	10 (8–17)	27 (19–35)	24 (15–32)	< 0.0001
Liver transplant and all‐cause mortality—median (IQR)				
30‐day mortality	None	40 (14)	40 (11)	< 0.0001
1‐year mortality	5 (7)	71 (25)	76 (22)	< 0.0001
1‐year liver transplant	8 (11)	107 (38)	115 (33)	< 0.0001

Abbreviation: TIPS: transjugular intrahepatic portosystemic shunt.

### 3.2 Comparison Between High‐ePVS Group (> 5.5) vs. Low‐ePVS Group (≤ 5.5)

Compared to the low e‐PVS group, the high‐ePVS group was younger (58 vs. 62 years, *p* = 0.0004) and had a lower proportion of males (57% vs. 71%, *p* = 0.028) and African Americans (26% vs. 40%).

### 3.3 Pulmonary Hemodynamics

On RHC, the high‐ePVS group had higher RAP (9 vs. 6 mmHg, *p* = 0.01), higher pulmonary arterial wedge pressure (PAWP) (14 vs. 11 mmHg, *p* = 0.014), higher CO (9.8 vs. 6.4 L/min, *p* < 0.0001), higher CI (5 vs. 3.1 L/min/m^2^, *p* < 0.0001), and lower pulmonary vascular resistance (PVR) (0.8 vs. 1.6 Wood Units, *p* < 0.0001).

### 3.4 Liver Disease Severity and Cirrhosis‐Related Complications

The median serum creatinine, INR, and total bilirubin levels were higher in the high‐ePVS group (*p* < 0.0001). In contrast, the serum sodium (136 vs. 139 mEq/L, *p* < 0.0001), total serum protein (6 vs. 6.9 g/dL, *p* < 0.0001), and serum albumin (2.9 vs. 3.3 g/dL, *p* = 0.001) were lower in the high‐ePVS group. The MELD‐Na score was higher in the high‐ePVS group (27 vs. 10, *p* < 0.0001) (Table 1). The high‐ePVS group had higher rates of cirrhosis‐related complications, including ascites (85% vs. 54%, *p* < 0.0001), splenomegaly (67% vs. 48%, *p* = 0.008), and a history of varices (42% vs. 19%, *p* < 0.0001).

### 3.5 30‐Day and 1‐Year Mortality Rates According to Liver Transplantation Status

Twenty‐two percent (*n* = 76) of LC patients died within a year after RHC (IQR 10–67 days), whereas 33% (*n* = 115) were transplanted within a year after RHC (IQR 5–52 days). Among transplant‐free patients, the high‐ePVS group had higher rates of 30‐day mortality (22% vs. 0%, *p* < 0.0001) and 1‐year mortality (36% vs. 8%, *p* < 0.0001). Meanwhile, among transplanted patients, 30‐day and 1‐year mortality were uncommon (2% and 7%, respectively) and no difference between ePVS groups was observed (Table 2).

**TABLE 2:** All‐cause 30‐day and 1‐year mortality stratified by estimated plasma volume status in transplanted and transplant‐free patients.

Variables	Low ePVS (≤ 5.5) (*n* = 72)	High ePVS (> 5.5) (*n* = 281)	Total (*n* = 353)	*p*
Transplant‐free patients (*n* = 238)				
30‐day mortality	None	38 (22)	38 (16)	< 0.0001
1‐year mortality	5 (8)	63 (36)	68 (29)	< 0.0001
Transplanted patients (*n* = 115)				
30‐day mortality	None	2 (2)	2 (2)	1
1‐year mortality	None	8 (7)	8 (7)	1

### 3.6 Survival Analysis

**FIGURE 1:**
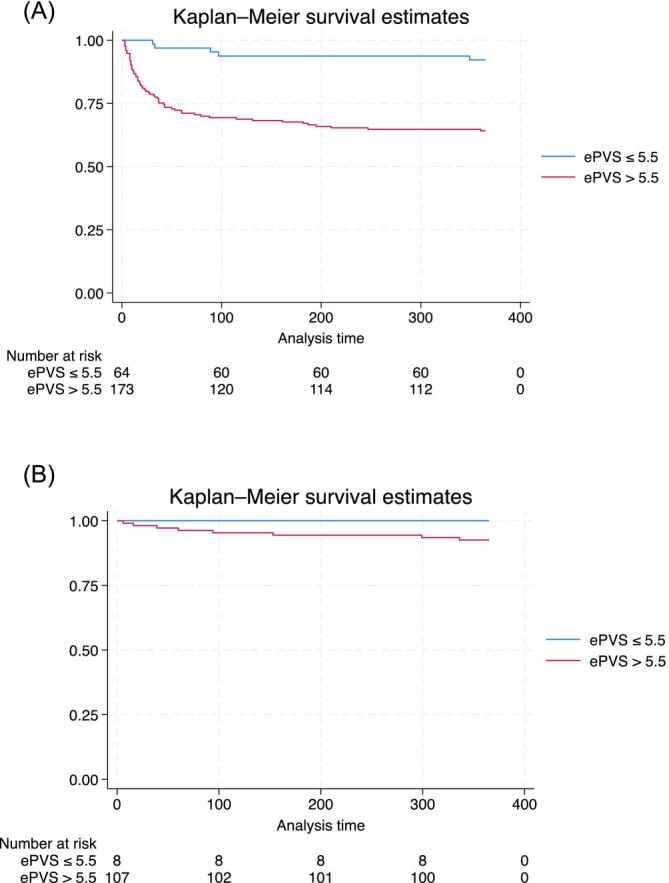
Kaplan–Meier curves of 1‐year all‐cause mortality after RHC stratified by high and low ePVS. (A) Transplant‐free patients (Log‐rank *p* < 0.0001). (B) Transplanted patients (Log‐rank *p* = 0.431).

On Kaplan Meier analysis, the high‐ePVS group had significantly lower 1‐year survival rates among transplant‐free patients (Log‐rank *p* < 0.0001) (Figure 1A). No difference between groups was observed among transplanted patients (Log‐rank *p* = 0.431) (Figure 1‐B). On Cox‐regression univariable analysis, higher ePVS was associated with all‐cause 1‐year mortality among transplant‐free patients (HR 1.38, CI 1.23–1.55, *p* < 0.0001). On Cox‐regression multivariable analysis, after adjusting for potential confounders, higher ePVS remained independently associated with all‐cause 1‐year mortality among transplant‐free patients (HR 1.15, CI 1.00–1.32, *p* = 0.048) (Table 3).

**TABLE 3:** Univariable and multivariable Cox regression analysis of factors associated with 1‐year mortality among transplant‐free patients.

Variables	Hazards ratio	95% confidence interval	*p*
Univariable analysis			
ePVS	1.38	1.23–1.55	< 0.0001
Multivariable analysis			
ePVS	1.15	1.00–1.32^a^	0.048
Age in years	1.02	1.00–1.05	0.043
Male sex	1.06	0.61–1.84	0.814
Model for end‐stage liver disease	1.09	1.06–1.12	< 0.0001
Serum albumin in mg/dL	0.83	0.62–1.13	0.248

^a^ Complete 95% confidence interval: 1.001336–1.326132.

### 3.7 Sensitivity Analysis

By using Youden's index method, the optimal ePVS cutoff for this study is 6.7. By using this cutoff, 34% of patients (*n* = 121) are reclassified as having low‐ePVS and 66% (*n* = 232) as high‐ePVS. In comparison, the high‐ePVS group continues to have higher cardiac filling pressures (RAP and PAWP), hyperdynamic circulation (higher CO and CI), more cirrhosis‐related complications (including ascites, splenomegaly, and varices), higher 1‐year liver transplant rates, and greater 30‐day and 1‐year all‐cause mortality (Table 4).

**TABLE 4:** Sensitivity analysis using estimated plasma volume status cutoff 6.7.

Variables—median (IQR)	Low ePVS (< 6.7) (*n* = 121)	High ePVS (≥ 6.7) (*n* = 232)	*p*
Right atrial pressure (mmHg)	7 (3–12)	9 (5–14)	0.032
Mean pulmonary artery pressure (mmHg)	23 (15–34)	24 (17–30)	0.832
Pulmonary arterial wedge pressure (mmHg)	12 (7–20)	14 (9–21)	0.031
Cardiac output (L/min)	6.8 (4.7–9)	11 (8–14)	< 0.0001
Cardiac index (L/min/m^2^)	3.4 (2.3–4.4)	5.4 (4.3–6.8)	< 0.0001
Pulmonary vascular resistance (Wood units)	1.5 (0.9–2.7)	0.8 (0.5–1.2)	< 0.0001
Ascites (*n* = 327)	64 (64)	198 (87)	< 0.0001
Splenomegaly (*n* = 327)	48 (48)	161 (71)	< 0.0001
Varices	28 (24)	103 (44)	< 0.0001
Portal venous circulation abnormalities	66 (64)	183 (82)	0.001
Model for end‐stage liver disease	13 (8–20)	26 (18–34)	< 0.0001
Model for end‐stage liver disease—Na	15 (9–22)	28 (20–35)	< 0.0001
30‐day mortality	2 (2)	38 (16)	< 0.0001
1‐year all‐cause mortality	10 (8)	66 (28)	< 0.0001
1‐year liver transplant	22 (18)	93 (40)	< 0.0001

## 4 Discussion

To our knowledge, this is the first study to apply the Strauss‐derived Duarte formula to categorize LC patients into high‐ and low‐ePVS groups. Our findings show that LC patients with high ePVS have more intravascular congestion, hyperdynamic circulation, and a higher prevalence of cirrhosis‐related complications. Additionally, we observed that ePVS is independently associated with all‐cause mortality at 1 year after adjusting for potential confounders among transplant‐free patients. These associations suggest that ePVS is a plausible prognostic marker in patients with cirrhosis with a meaningful physiologic substrate.

When comparing the high and low‐ePVS groups, the high‐ePVS group exhibited signs of hyperdynamic circulation, a state frequently observed in advanced liver disease, including higher CO, CI, and lower PVR [10]. These changes reflect a heightened vasodilatory state among the high‐ePVS group likely driven by elevated levels of circulating vasoactive mediators, such as nitric oxide [11]. Hyperdynamic circulation is characteristic of advanced liver disease and portal hypertension and is associated with worse clinical outcomes owing to its associated complications, including ascites, variceal bleeding, and renal dysfunction [12]. In our study, these cirrhosis complications were more common among the high‐ePVS group as well.

Furthermore, the high‐ePVS group had higher right‐ and left‐sided filling pressures, including RAP and PAWP. Intravascular congestion is a known complication seen in advanced liver disease [13], and its association with high ePVS is likely secondary to plasma volume expansion. Initially, LC patients present with low effective intravascular volume as a result of hypoalbuminemia and third spacing [14]. Low effective intravascular volume, along with systemic vasodilation (due to portal hypertension) and increased intraabdominal pressures (due to ascites), causes renal hypoperfusion and subsequent activation of the renin‐angiotensin‐aldosterone axis, leading to sodium and water retention [15]. Meanwhile, antidiuretic hormone stimulation and release also contribute to hypervolemia and hyponatremia in LC patients [11, 16, 17, 18, 19, 20].

In terms of outcomes, our study revealed that higher ePVS is associated with all‐cause mortality at 1 year in transplant‐free patients after adjusting for potential confounders. Supporting evidence from other studies highlights the prognostic value of ePVS in various clinical settings [21, 22, 23]. For instance, in HF patients, a higher ePVS has been associated with increased mortality and adverse cardiovascular events [24]. Duarte et al. showed that an elevated ePVS was a strong predictor of mortality in patients with chronic HF following acute myocardial infarction [25]. Likewise, Chen et al. found that higher ePVS was associated with major cardiovascular events and increased mortality in patients with acute myocardial infarction [26].

In liver cirrhosis, a study recently published by Nørskov et al. compared ePVS to the dilution technique as the gold‐standard method (PVI‐125) to determine the volume status. They found that although ePVS correlated significantly with actual plasma volume, there were wide limits of confidence, thereby concluding that using it as a surrogate of volume status for clinical practice would not be appropriate [27]. However, this has been true for other well‐studied diseases, such as acute HF [28] but ePVS still showed a significant correlation with prognosis [25] and other objective data of volume overload [3], like it did in our study.

Of note, the higher rate of OLT in patients with high ePVS likely reflects their more advanced liver disease and greater burden of complications owing to higher MELD‐Na scores, hyperdynamic circulation, and vascular congestion [29]. However, the 1‐year mortality rate among transplanted patients was exceedingly low, which reflects the substantial clinical benefit of liver transplantation among LC patients with hemodynamic alterations. As expected, ePVS was not associated with adverse outcomes among transplanted patients. Successful liver transplantation results in normalized hemodynamics [30], likely impacting the prognostic value of ePVS during the pre‐transplant period.

Given the association of high‐ePVS with intravascular congestion and hyperdynamic circulation, risk stratifying LC patients using ePVS in addition to MELD‐Na in clinical practice may help identify LC patients with early circulatory dysfunction, even if their MELD‐Na score is not critically high. Those with higher ePVS may benefit from closer monitoring and optimized diuretic therapy to prevent volume overload. Furthermore, patients with high‐ePVS and refractory ascites or recurrent variceal bleeding could be considered for earlier TIPS placement. Lastly, combining MELD‐Na and ePVS may help refine *organ allocation* by identifying *patients at high risk of mortality despite moderate MELD*‐*Na scores* [31]. Among heart failure patients, there is an ongoing effort to use ePVS as a longitudinal assessment for volume status monitoring [32]. Likewise, longitudinal ePVS measurements could play a dynamic role in the risk stratification of LC patients, allowing for *earlier interventions* before complications arise [32].

This study had several important limitations. First, as a retrospective analysis, it is inherently vulnerable to biases typical of observational data, including selection bias and unmeasured confounding variables, which may have influenced the observed associations. Second, ePVS was measured only once, which restricts our understanding of how fluctuations in ePVS over time may correlate with disease progression or clinical outcomes. Regular, longitudinal measurements of ePVS could provide valuable insights into the dynamic nature of cirrhosis and its response to therapeutic interventions [33]. Similarly, RHC data represents only a snapshot of patients' hemodynamics over time. Although cardiac filling pressures (including RAP and PAWP) were different between high‐ and low‐ePVS groups, we acknowledge there is a substantial overlap between groups. Likewise, we acknowledge that the association of ePVS and all‐cause mortality could range from no effect to a modest increase in mortality risk (CI 1.00–1.32). Therefore, future studies with larger sample sizes may clarify this relationship, potentially revealing a stronger association if one exists. Finally, future prospective studies are needed to better clarify the mechanisms behind elevated ePVS in cirrhotic patients and their role in prognostication and clinical management.

## 5 Conclusion

Among patients with liver cirrhosis, high ePVS was associated with hyperdynamic circulation, intravascular congestion, and cirrhosis complications. Furthermore, high ePVS is independently associated with all‐cause mortality among transplant‐free patients. This suggests that ePVS may serve as a valuable prognostic marker in LC patients. Further research is needed to clarify its role in guiding clinical management and risk stratification.

## Ethics Statement

This study was approved by Jefferson's institutional review board (iRISID‐2023‐2277).

## Consent

No patient consent was needed due to retrospective data without identifiers with approval from Jefferson's institutional review board (iRISID‐2023‐2277).

## Conflicts of Interest

The authors declare no conflicts of interest.

## Data Availability

The data that support the findings of this study are available from the corresponding author upon reasonable request.
